# Dropout Analysis of a National Social Health Insurance Program at Pokhara Metropolitan City, Kaski, Nepal

**DOI:** 10.34172/ijhpm.2021.171

**Published:** 2021-12-14

**Authors:** Prabin Sharma, Dipendra Kumar Yadav, Niranjan Shrestha, Prabesh Ghimire

**Affiliations:** ^1^School of Health and Allied Sciences, Pokhara University, Pokhara, Nepal.; ^2^Central Department of Public Health, Institute of Medicine, Tribhuwan University, Kathmandu, Nepal.

**Keywords:** Insurance, Social Health Insurance, Health Insurance Board, Dropout, Nepal, Pokhara

## Abstract

**Background:** Nepal’s national social health insurance (SHI) program, which started in 2016, aims to achieve universal health coverage (UHC), but it faces severe challenges in achieving adequate population coverage. By 2018, enrolment and dropout rates for the scheme were 9% and 38% respectively. Despite government’s efforts, retaining the members in SHI scheme remains a significant challenge. The current study therefore aimed to assess the factors associated with SHI program dropout in Pokhara, Nepal.

**Methods:** A cross-sectional household survey of 355 households enrolled for at least one year in the national SHI program was conducted. Face-to-face interviews with household heads were conducted using a structured questionnaire. Data was entered in Epi-Data and analysed using SPSS. The factors associated with SHI program dropout were identified using bivariate and multiple logistic regression analyses.

**Results:** The findings of the study revealed a dropout prevalence of 28.2% (95% CI: 23.6%-33.2%). Households having more than five members (adjusted odds ratio [aOR]: 2.19, 95% CI: 1.22-3.94), belonging to underprivileged ethnic groups (Dalit/Janajati) (aOR: 2.36, 95% CI: 1.08-5.17), living on rented homes (aOR: 4.53, 95% CI: 1.87-10.95), absence of chronic illness in family (aOR: 1.95, 95% CI: 1.07-3.59), perceived good health status of the family (aOR: 4.21, 95% CI: 1.21-14.65), having private health facility as first contact point (aOR: 3.75, 95% CI: 1.93-7.27), poor availability of drugs (aOR: 4.75, 95% CI: 1.19-18.95) and perceived unfriendly behaviour of service providers (aOR: 3.09, 95% CI: 1.01-9.49) were statistically significant factors associated with SHI dropout.

**Conclusion:** In Pokhara, more than one-fourth of households have dropped out of the SHI scheme, which is a significant number. Dropping out of SHI is most commonly associated with a lack of drugs, followed by rental housing, family members’ reported good health status and unfriendly service provider behaviour. Efforts to reduce SHI dropout must focus on addressing drugs availability issues and improving providers’ behaviour towards scheme holders. Increasing insurance awareness, including provisions to change first contact points, may help to reduce dropouts among rented households, which make up a sizable proportion of the Pokhara metropolitan area.

## Background

**Key Messages**

** Implications for policy makers**

- The health insurance board (HIB) may need to work with health facilities as well as federal, provincial and local governments, to improve the availability of all drugs listed in the benefit package. Adding pharmacy services to the health facilities, increasing public hospital budgets and accelerating the procurement process, could all help to improve drug availability.
- In this study, more than a quarter of households who dropped out of the social health insurance (SHI) mentioned a lack of awareness about renewal mechanisms as their reason for not continuing their scheme. The adoption of digital innovations such as insurance policy renewal reminder service or text message notifications, as well as proactive involvement of enrolment assistant in informing health insurance subscribers about renewal procedures may help increase members’ renewal in the SHI.
- Because the primary goal of the SHI is to provide financial protection to everyone from future risks of health-care costs, the HIB must adopt more pragmatic methods of retaining even the low-risk households in the SHI scheme. To advance towards universal health coverage (UHC), Nepal should replace voluntary financing with mandatory enrolment by enforcing the criteria under the Health Insurance Act and also use tax financing to cover informal sectors and households.

** Implications for the public**

A significant number of households in the metropolitan area are rented, and retaining them in the scheme is critical to the program’s long-term success. Despite the fact that such households had the option of changing their first point of contact, the increased likelihood of rented households dropping out of social health insurance (SHI) was unexpected. Focused community-based awareness and information dissemination on SHI provisions, including policies and processes for changing first contact points, are necessary to ensure their retention and expanded coverage. On the supply side, unfriendly provider behaviour has been found to influence SHI dropout. Health workers may need to cultivate a positive attitude toward health insurance members. This could include giving clients a warm welcome, paying attention and respect, taking a polite approach, providing adequate and relevant health information, and treating insured and uninsured members equally.

Social health insurance (SHI) is one of the principal methods of health financing. A number of countries around the world have already achieved universal health coverage (UHC) using this method, and a few others have come close.^1,2^ In recent years, several low-and middle-income countries including Nepal have embarked on a path to implement various models of health insurance schemes, aiming to cover their entire population. In 2016, the Government of Nepal initiated a SHI program with the objective to ensure UHC by increasing access to and utilization of necessary quality health services. The Health Insurance Act of 2017 and its regulations of 2019, guide the implementation of this program, which is currently administered by the Health Insurance Board (HIB).^3^

Reaching UHC through health insurance is a difficult process that necessitates careful considerations of numerous factors. The program’s success is dependent on its ability to achieve high levels of geographical, population and service coverage. Nonetheless, the existing evidence show that the national SHI program in Nepal has encountered a number of challenges in achieving an adequate population coverage.^4^ By 2018, the SHI program had expanded to 36 districts, with more than 1.5 million members enrolled.^5^ While this enrolment represented 9% of the total population, the dropout rate was 38%. The rate of dropping out of the health insurance program varied by district, ranging from 15% to 96%.^4^ Despite the government’s efforts, ensuring the SHI scheme’s long-term viability remains a significant challenge. Low enrolment and higher dropout rates frequently undermine the government’s goal of providing financial protection and access to quality healthcare for its citizens.

Globally, a large body of literature has investigated the factors that influence health insurance dropout. Higher dropout rates have been predominantly linked to high premiums, unfriendly provider behaviour, limited scheme benefits, poor quality of care, and a lack of trust in contracted heath facilities and health insurance schemes.^6-12^ While the dropout problems are persistent in many countries, many of the contributing factors appear to be highly country and context-specific depending on the modalities of the insurance program, the characteristics of the health system, and the socioeconomic circumstances and conditions of the target population in each country. For instance, while non-affordability of the premium was the main reason for dropout in Ghana’s health insurance scheme, poor perceived health service quality, failure to provide the promised benefit package, and providers’ perceived negative attitude toward insured members were all significant contributing determinants of high dropout in the community-based health insurance (CBHI) scheme in Manna district, Ethiopia.^13^ Similarly, perception of poor quality of health services was the most important determinant of drop-out in Senegal’s CBHI scheme. The current study is therefore justified by the need to understand the dropout determinants that are unique to Nepal’s SHI context.

Despite this need, Nepal’s health insurance scheme has a weaker evidence base, with even fewer studies on the factors that influence SHI enrolment^14,15^ and dropouts.^4,10^ Small-scale studies on SHI enrolment have already established that the ethnically privileged and well-off families and those suffering from acute and chronic health conditions are more likely to enrol in the Nepal’s SHI scheme.^14,15^ Policy-makers anticipate that the same factors that influence enrolment will also influence renewal and dropout decisions. If that were the case, everyone who were enrolled to the scheme would indeed renew their membership. The factors that influence renewal and dropout decisions are distinct from those that influence enrolment decisions.

Nonetheless, only a few studies have looked into the factors that lead to SHI dropout in Nepal. The literature suggests that SHI in Nepal has failed to satisfy its scheme holders due to factors such as drug shortages, insufficient benefit package content, poor quality of health services, and unfriendly behaviour of health workers.^4^ Furthermore, larger families as well as those who are more affluent and ethnically privileged are the ones with greater likelihood of dropping out.^4,14,16^ In the study conducted in Pokhara-Metropolitan area, the authors attempted to confirm an association with certain socio-demographic and service related factors, but the dropout factors were not clearly established.^10^ On the other hand, this study overlooked many important factors related to health service and scheme characteristics, such as service quality, availability of drugs and services promised by the SHI scheme, service providers’ behaviour, and premium affordability, all of which could be potential factors for SHI dropout. Another study attempted to take into account many of these factors, but the results were limited by the exploratory nature of the study.^4^

 In this background, the current study used a quantitative methodology with a robust design to corroborate the range of factors identified by previous studies as well as to identify additional factors that have not yet been investigated for Nepal. The aim is to provide the HIB, as well as other policy and decision makers, with the information they need to devise policies that would improve members’ retention in the SHI, thereby ensuring the program’s long-term viability in Pokhara and throughout the country. This article is expected to contribute to the entire knowledge base of the health insurance program in Nepal by identifying the factors related with dropout in Nepal’s SHI program.

###  Features of Nepal’s Social Health Insurance Program

The Government of Nepal in 2016 introduced a SHI scheme called Social Health Security Program (SHSP) aiming to provide an affordable financial protection against the risk of health-related costs.^17^ Social Health Security Development Committee, a semi-autonomous body initially administered this program. The first phase of the SHSP was implemented in three districts – Kailali, Baglung and Ilam and was gradually expanded to other districts.^3,17^ After the Health Insurance Act was endorsed in 2017, SHSP was renamed as Health Insurance Program and the autonomous HIB formed under the provision of the act has been administering the program thereafter. The program currently aims to cover all households, families of civil servants, formal enterprises and even the persons going abroad for foreign employment. Although the Health Insurance Act, 2017 makes it mandatory for every Nepali citizen to enrol in the health insurance program, the SHI is still voluntary in practice due to lack of incentives and processes to register the informal sector.^3^

The SHI in Nepal receives funds from two main sources: pre-paid premiums (membership contributions) and public funds, which are Government of Nepal revenues as annual block grant directed to the health insurance fund to subsidize the premiums for targeted groups and to cover its administrative expenses.^3^ As a prepaid premium, families up to five members contribute Nepali Rupees (NPR) 3500 per year (US$29.37) and NPR 700 (US$5.87) for each additional member in the family.^18^ Enrolment assistants collect the premium contributions from households. In case of formal sector, premium contribution has been set at 2% of the basic salary not exceeding NPR 10 000 (US$83.92) per year (1% contribution each by the employee and employer).^18^ There are policy provisions for exemptions in annual premium such that government of Nepal pays certain premiums for the definite group of people. The exemption rates are 100% for the families of ultra-poor, severe disabled, leprosy, HIV infected and complicated tuberculosis (TB) cases (multidrug-resistant-TB), 100% for senior citizens aged 70 years or above and 50% for female community health volunteers (Table 1).^18^

**Table 1:** Features of Nepal’s Social Health Insurance Program

**Features**	**Description**
Roll out year	April 2016. The program was rolled out in Pokhara (Kaski district) from December 2016. At the time of the study, the program was being rolled out in 42 districts; 30 districts had completed their 1-year enrolment cycle.
Administration	Administered by HIB, an autonomous body established under Health Insurance Act, 2018. The board provides membership cards, contracts and negotiates with service providers on benefit package, their costs and deals with provider payments.
Sources of revenue	Budget allocated by Government of Nepal. Premium contributions from households where families with up to five members contribute NPR 3500 (US$29.37^a^) per year and NPR 700 (US$5.87) per additional member. 2% payroll contribution from formal sector not exceeding NPR 10 000 (US$839.20) per year (1% contribution each by the employee and employer).
Exemptions	100% exemption in annual premium for families of ultra-poor, severe disability, leprosy, HIV infected, complicated TB cases (MDR-TB). 100% exemption for senior citizens aged 70 years or above. 50% exemption for female community health volunteers.
Pooling arrangements	Single pool at national level managed by the HIB.
Service delivery channels	Public health facilities as first point of contact. Private health facilities selected through contracting - for emergency and referral services.
Benefit Package	Benefits of up to NPR 100 000 per year are provided to insured families of up to five members with an additional NPR 20 000 (US$167.84) covered for each additional member. The maximum amount of benefit available to a family per year is NPR 200 000 (US$1678.41).^18^ Benefits of up to NPR 100 000 per year to be utilized only by senior citizens aged 70 years or above. Additional NPR 100 000 for treatment of cancer, cardiovascular disease, kidney disease, head injury and spinal injury, sickle cell anemia, Parkinson disease and Alzheimer disease. Covers emergency services, outpatient consultations, inpatient services, surgeries, drugs, and laboratory tests, radiological and other diagnostic services. Includes over 1000 types of medicines including 25 types of ayurvedic medicines.^19^
Co-payments	No co-payments or other cost sharing arrangements in practice.
Exclusion list	Ambulance services other than during emergency. Plastic and cosmetic surgeries except for treatment related to burns, severe disability and surgery for cleft lip and palate. Spectacles, hearing aids, white canes and crutches costing more than the price determined by the HIB. Dental services except extraction, draining of abscess and primary management of dental injuries (trauma).^18^
Provider payment mechanisms	Case-based payment for outpatient and emergency services. Fee for service for inpatient and diagnostic services.
Renewal	Families need to renew their membership one month prior to the end of membership period. Families that are not able to utilize any amount from the benefit package are offered 10% discount in the annual premium during the renewal for next year.

Abbreviations: HIB, Health Insurance Board; MDR-TB, multi-drug resistant tuberculosis; NPR, Nepali Rupees; TB, tuberculosis. ^a^ 1 USD equivalent to NPR 119.16 based on exchange rate of Nepal Rastra Bank for July 30, 2021.

The SHI provides coverage for emergency services, outpatient consultations, inpatient services, surgeries, drugs, and laboratory tests, radiological and other diagnostic services. The services are provided through both public and private facilities (contracted) with some specialized curative care at selected super-specialized and tertiary hospitals, and public providers are the first point of contact.^3^ The benefit package includes over 1000 types of allopathic and ayurvedic medicines.^19^ Each insured households of up to five members receives a benefits of up to NPR 100 000 (US$839.20) per year while an additional NPR 20 000 (US$167.84) is covered for each additional member. The maximum amount of benefit available to a family per year is NPR 200 000 (US$1678.41).^18^ However, senior citizens aged 70 years or above receives an additional benefit of up to NPR 100 000 per year. In case of members suffering from cancer, cardiovascular disease, renal failure, head and spinal injury, sickle cell anemia, Parkinson disease and Alzheimer disease, additional benefit of up to NPR 100 000 is provided for the treatment of these diseases.^19^

SHI also has a list of conditions that are excluded in the benefits package. These includes ambulance services except for specific conditions during emergency, plastic and cosmetic surgeries other than the treatment related to burns, severe disability and surgery for cleft lip and palate. Other items in the exclusion list includes optical aids, hearing aids, orthopaedic aids (white canes and crutches) costing more than the price determined by the HIB, dentures and dental services except extraction, draining of abscess and primary management of dental injuries (trauma).^18^ The payments in SHI program are made directly to the service provider organizations. A number of different provider payment systems such as fee for services, case-based payments and capitation are used to purchase health services.^3^ Provider payment is streamlined through electronic Insurance Management Information System. Families who complete their one year of enrolment need to renew their membership one month prior to the end of membership period. Families that are not able to utilize any amount from the benefit package during the one-year enrolment cycle are offered 10% discount in the annual premium during the renewal in the subsequent year (Table 1).^18^

## Methods

###  Study Setting

The SHI scheme was being implemented in 42 districts at the start of this study, with 30 districts having completed their one-year program rollout and eight districts having completed their two-year program rollout. Pokhara metropolitan city in Kaski district was chosen as the most convenient of the eight districts. Pokhara is one of Nepal’s six metropolitan cities and the provincial capital of the Gandaki province. Administratively, it is divided into 33 wards (lowest administrative unit). Pokhara metropolitan city is located approximately 204 km (126 miles) west of Kathmandu, the country’s capital city. The city has a total area of 464.24 km^2^ and a population of 414 141 people.^20^ Pokhara is one of the most urbanized areas in Nepal, with 55% of households living in rented homes. The SHI program in Pokhara metropolitan (Kaski district) began in December 2016. At the time of the study, 18 807 (4.5%) households were enrolled in the SHI program with 13 339 having completed one year of coverage.^21^ In Pokhara, eight health facilities were designated to provide health services to the insured populations, three of which were public sector health facilities.

###  Study Design and Population

 A community-based cross-sectional analytical study was conducted among households that had been members of SHI for at least a year. Households enrolled in the SHI between December 16, 2016 and January 14, 2018 were eligible for inclusion in the study.

###  Sample Size and Sampling Procedure

The sample size was calculated using a single population proportion formula for finite population^22^ assuming the proportion of households dropping out from the SHI (p) at 38.5%,^21^ a precision (d) of ±5% and a standard normal variate (Z_α/2_) of 1.96 at 5% significance level. Using the finite population (N) of 13 339 (households in Pokhara metropolitan city that had completed one year of enrolment in SHI), the estimated sample size was 355.


$$Sample\;size\left(n\right)=\frac{NZ_{\alpha /2}{}^{2}p\left(1-p\right)}{d^{2}\left(N-1\right)+Z_{\alpha /2}{}^{2}p\left(1-p\right)}$$

The list of households that had completed one year of enrolment was obtained from the HIB. Our sample was drawn at random from 11 wards in the Pokhara metropolitan area. The sample size in each ward was proportional to the number of insured households in that ward who met the inclusion criteria. A simple random sampling technique was used to sample households in each ward. This was accomplished by computer randomization of eligible households. Ward office (lowest administrative level of the local government) personnel and enrolment assistants (those responsible for registering and enrolling families in the SHI) helped in locating the sampled households.

###  Variables

The outcome variable in this study was current SHI status (drop-out or continuous use). We used the Andersen’s behavioural model of health-care utilization^23,24^ as a framework for the explanatory variables to predict the factors that facilitate or hinder households to continue their SHI membership or dropout out of the scheme. This model suggests that the utilization or non-utilization of healthcare and services is determined by the three key factors (predisposing, enabling and need factors).^23^ The selection of explanatory variables was guided by review of literatures on the determinants of dropout from the health insurance schemes.^4,7,8,11,13,25-28^ In this study, the predisposing factors included socio-demographic characteristics (age, sex, household size, and ethnicity), knowledge, and attitude towards SHI. The economic factors such as home ownership, occupation and wealth index of household was examined as enabling factors. Other enabling factors included type of health facility as first contact point, distance from health facility (first contact point), time to reach health facility, waiting time, availability of service providers, availability of services under benefit package, perceived quality of services, perceived behaviour of service providers and satisfaction with opening hours. The need factors in this study included presence of dependent members (children less than 18 years and elderly people above 60 years of age) in the family, past illness experience, presence of chronic illness in the family, perceived family health status and presence of differently abled member in the family.

###  Operational Definition

*Dropout from SHI:* Households that had previously been members of SHI but were no longer enrolled (at the time of the survey) were classified as dropouts. Households that dropped out of the SHI were coded as 1 and 0 otherwise.

*Continuous use:* Households that had been a member of SHI for more than a year and still had a valid membership at the time of survey, as well as households that had been enrolled for a year and then renewed their membership after a certain number of months of discontinuation but still had a valid membership at the time of survey were considered as “continuous user.”

*Knowledge on SHI:* Household heads’ knowledge of SHI was assessed using 14-item knowledge questions about annual premium, annual benefit amount and membership renewal time. A score of one was assigned to each correct answer. Based on the total score obtained by the respondent, the overall knowledge of SHI was divided into three categories: poor knowledge (50% score), moderate knowledge (50%-75% score) and good knowledge (>75% score).

*Attitude towards SHI:* The attitude of the household heads was assessed using a 5-point Likert scale ranging from strongly disagree (1 point) to strongly agree (5 points). Eleven positive and negative statements about SHI were used to better understand the respondents’ perspectives. The scores for the negative statements were reversed. Hence, the highest score that the respondents could obtain was 55 and their lowest possible score was 11. Based on the points obtained, respondent’s attitude was graded as either favorable (scoring more than or equal to 33), or unfavourable (scoring less than 33).

*Household wealth:* An international wealth index (IWI) was used to assess the wealth status of households. IWI is an asset-based wealth index that ranges from 0 to 100, with 0 representing households with no assets and 100 representing households having all assets and the highest quality housing.^29^ In order to calculate this, data on household durables, access to basic services, and housing characteristics were entered into the IWI formula.^30^ The elderly participants were divided into five wealth quintiles based on their IWI score, with the first quintile representing the poorest segment of the population and the fifth quintile representing the least poor.

*Illness experience in the family after membership:* The status of past illness experience was recorded by asking the respondents to recall if any of their family members had become ill after joining SHI. The responses were either ‘Yes’ or ‘No.’

*Presence of family member with chronic illness:* The presence of chronic illness was recorded as “Yes” if any member in the family had been constantly taking medicines for the past three months or is required to take medicines for more than three months for any medically confirmed chronic condition.

*Perceived family health status:* This was a self-rated response measured on a five-point Likert scale ranging from excellent to poor. The data was later categorized as good or poor.

###  Data Collection 

Data collection was carried out using a structured questionnaire. The choice of questions in the instrument was guided by the review of literatures on enrolment and dropout in health insurance schemes.^7-9,14,25-27,31^ Suggestions and feedback on the content of the tool was obtained from the research supervisors and experts and their comments were incorporated in the final version of the instrument. The questionnaire was initially prepared in English and then translated into the Nepali language for ease of administration. Prior to application, the questionnaire was pretested in 10% of the sample outside the study area and changes were made based on the pretest results. Cronbach’s alpha was used to determine the internal consistency of items on the attitude scale (five-point Likert scale). The scale’s alpha value was 0.69, which was close to the commonly accepted value of 0.70.^32^ Hence, it was accepted. The data was collected between May and June of 2019. Face-to-face interview with the household head (household member who was the key income provider and primary decision-maker in the family) was used to collect data. In case of unavailability of the household head during the visit, the next closest household meeting the inclusion criteria was chosen for interview. Data collection was carried out by the first author and the two enumerators. The enumerators had a university degree in public health and were familiar with the interview procedure and information sought. To ensure quality, the collected data was thoroughly reviewed for clarity and completeness.

###  Data Management and Analysis

The collected data were entered, cleaned, coded and consistency were checked using EpiData version 3.1. The coded data was then exported and analyzed with SPSS version 20. The background characteristics of the study population was examined using univariate analysis. For continuous variables, we calculated measures of central tendency and dispersion, and for categorical variables, we calculated proportions. Binary logistic regression was used to estimate independent associations between explanatory variables and the binary outcome variable. All explanatory variables with *P* value <.15 in the bivariate analysis were included in the multiple logistic regression model. This was done to eliminate any variables that could potentially make a significant contribution in multiple regression analysis in the presence of other variables. Variables were tested for multicollinearity before being entered into the regression analysis. Using collinearity diagnostics, one variable, “used SHI card after membership,” was found to be multicollinear (variance inflation factor >10) and was thus excluded from the analysis. The predicted value was described using adjusted odd ratios (aORs) (estimated by taking the antilog of the logistic coefficient) at 5% level of significance. The regression model’s goodness of fit was determined using the Hosmer and Lemeshow chi-square test. The model was found to be a good fit with *P*> .05. In addition, a Nagelkerke (pseudo) R square value of 0.403 was observed in the model.

 The following equation explained the regression model:

*Log [y/(1-y)] = β_0 _+ β_1_x_1_ + β_2_x_2_+ β_3_x_3_+…… + β_n_x_n_ + e*

Where ‘y’ is the expected probability of the outcome variable occurring, b_0_ is the constant or intercept, β_1_, β_2_, β_3 _... β_n _are the regression coefficients, x_1_, x_2_, x_3 _... x_n_ are *n* independent variables, and *e* is the error term.

## Results

###  Characteristics of the Study Population 

The mean age of the respondents was 50.3 ± 12.4 years. Almost two-thirds (65.1%) household were headed by males. More than three in five households (63.4%) had equal to or less than five members. The average household size of the study population was 5.4 ± 1.3. A greater majority of the households (85.4%) belonged to privileged ethnic group (Brahmin/Chhetri) and lived in housed owned by either themselves or a family member (88.5%). Just more than half (51.0%) households were employed in the paid sector (business, labor, service and foreign employment). While just more than a fourth (26.8%) of the study participants had a poor knowledge on SHI, about three in five (58.9%) had an unfavorable attitude towards SHI. More than four in five households (82.3%) had at least one dependent member in the family. A greater majority of the households (91%) had an experience of illness in the family after their membership in SHI, whereas nearly half (46.5%) had a family member suffering from chronic illness. Slightly more than one in ten household heads (11.5%) perceived the health status of their family as poor (Table 2).

**Table 2:** Socio-demographic, Economic, Morbidity Status, Health Service and Health Insurance Scheme Characteristics Among Households in Pokhara Metropolitan City, Kaski District, Nepal (n = 355)

**Variables**	**SHI Status of Households**		**Total**
	**Dropout ** **No. (%) (n = 100)**	**Continuous Use** **No. (%) (n = 255)**	
Age of household head^a^			
Less than 40 years	23 (23.0)	44 (17.3)	67 (18.9)
40-59 years	58 (58.0)	155 (60.8)	213 (60.0)
60 years or older	19 (19.0)	56 (22.0)	75 (21.1)
Gender of household head			
Male	54 (54.0)	177 (69.4)	231 (65.1)
Female	46 (46.0)	78 (30.6)	124 (34.9)
Household size^b^			
Less than or equal to 5 members	54 (54.0)	171 (67.1)	225 (63.4)
More than 5 members	46 (46.0)	84 (32.9)	130 (36.6)
Ethnicity			
Privileged (Brahmin/Chhetri)	80 (80.0)	223 (87.5)	303 (85.4)
Underprivileged (Dalit/Janajati)	20 (20.0)	32 (12.5)	52 (14.6)
Home ownership			
Self-owned	79 (79.0)	235 (92.2)	314 (88.5)
Rented	21 (21.0)	20 (7.8)	41 (11.5)
Main occupation of the household			
Unpaid work (agriculture and home maker)	50 (50.0)	124 (48.6)	174 (49.0)
Paid work (Business, service, labor and foreign employment)	50 (50.0)	131 (51.4)	181 (51.0)
Wealth index			
Q1-Poorest	21 (21.0)	51 (20.0)	72 (20.3)
Q2-Poor	25 (25.0)	45 (17.6)	70 (19.7)
Q3-Middle	19 (19.0)	54 (21.2)	73 (20.6)
Q4-Rich	16 (16.0)	61 (23.9)	77 (21.7)
Q5-Richest	19 (19.0)	44 (17.3)	63 (17.7)
Knowledge of household head on SHI			
Poor	34 (34.0)	61 (23.9)	95 (26.8)
Moderate	41 (41.0)	115 (45.1)	156 (43.9)
Good	25 (25.0)	79 (31.0)	104 (29.3)
The attitude of the household head towards SHI			
Unfavorable	63 (30.1)	146 (69.9)	209 (58.9)
Favorable	37 (25.3)	109 (74.7)	146 (41.1)
Dependent members in the family			
Yes	84 (84.0)	208 (81.6)	292 (82.3)
No	16 (16.0)	47 (18.4)	63 (17.7)
Illness experience in the family after membership			
Yes	89 (89.0)	234 (91.8)	323 (91.0)
No	11 (11.0)	21 (8.2)	32 (9.0)
Presence of family member with chronic illness			
Yes	35 (35.0)	130 (51.0)	165 (46.5)
No	65 (65.0)	125 (49.0)	190 (53.5)
Perceived health status of a family by the household head			
Good	95 (95.0)	220 (86.3)	315 (88.7)
Poor	5 (5.0)	35 (13.7)	40 (11.3)
Health facility as a first contact point			
Public	52(52.0)	179 (70.2)	231 (65.1)
Private	48 (48.0)	76 (29.8)	124 (34.9)
Used SHI card to access service benefits			
Yes	67 (67.0)	220 (86.3)	287 (80.8)
No	33 (33.0)	35 (13.7)	68 (19.2)
Distance to reach health facility (first contact point) for SHI services (n = 287)			
<30 min	13 (19.4)	49 (22.3)	62 (21.6)
≥30 min	54 (80.6)	171 (77.7)	225 (78.4)
Waiting time (n = 287)			
<30 min	8 (11.9)	9 (4.1)	17 (5.9)
30-60 min	29 (43.3)	68 (30.9)	97 (33.8)
>60 min	30 (44.8)	143 (65.0)	173 (60.3)
Availability of service provider (n = 287)			
Frequently available	61 (91.0)	205 (93.2)	266 (92.7)
Not available frequently	6 (9.0)	15 (6.8)	21 (7.3)
Availability of services under SHI (n = 287)			
Frequently available	29 (43.3)	137 (62.3)	166 (57.8)
Not available frequently	38 (56.7)	83 (37.7)	121 (42.2)
Availability of drugs under SHI (n = 287)			
Frequently available	3 (4.5)	54 (24.5)	57 (19.9)
Not available frequently	64 (95.5)	166 (75.5)	230 (80.1)
Perceived behaviour of service providers (n = 287)			
Friendly	11(16.4)	85 (38.6)	96 (33.4)
Average	35 (52.2)	107 (48.6)	142 (49.5)
Not friendly	21 (31.3)	28 (12.7)	49 (17.1)
Perceived quality of services (n = 287)			
Satisfactory	48 (71.6)	191 (86.8)	239 (83.3)
Not satisfactory	19 (28.4)	29 (13.2)	48 (16.7)
Satisfaction with opening hour of HF (n = 287)			
Satisfactory	12 (17.9)	80 (36.4)	92 (32.1)
Not satisfactory	55 (82.1)	140 (63.6)	195 (67.9)
Affordability of premium			
Affordable	67 (67.0)	203 (79.6)	270 (76.1)
Difficult to afford	33 (33.0)	52 (20.4)	85 (23.9)

Abbreviation: SHI, social health insurance. ^a^Mean ± standard deviation (SD) = 50.3 ± 12.4 years; ^b^Mean ± SD = 5.4 ± 1.3 and minimum-maximum household size = 3-11.

Similarly, about two-third households (65.1%) enrolled in SHI had public health facility as a first contact point. More than four in five households (80.8%) had used the SHI card to access the health services available in the benefits package. Among those utilizing the service benefit package, only one in five households (78.4%) had their first contact point (health facility) at a distance of less than 30 minutes. The maximum time to reach the first contact point was 150 minutes. About three in five participants (60.3%) reported that they had to wait for more than an hour to receive the services under SHI. While the greater majority of respondents (92.7%) reported that the service providers were regularly available at the health facility at the time of their visit, only less than three in five (57.8%) reported that the services under benefit package was regularly available. Only about two in five respondents (19.9%) reported that the drugs under SHI were frequently available. Less than two in five (17.4%) respondents perceived that the behaviour of health service providers was not friendly. Similar proportion of the respondents (16.7%) were not satisfied with the quality of health services. Likewise, more than two-third respondents (67.9%) were not satisfied with the opening hours of the health facility. About a fourth (23.9%) of the respondents stated difficulty to afford the annual premium of SHI (Table 2).

###  Magnitude and Reasons of Dropout From Social Health Insurance Program 

More than a fourth (28.2%, 95% CI: 23.6%-33.2%) of the households had dropped out from the SHI program (Figure).

**Figure:**
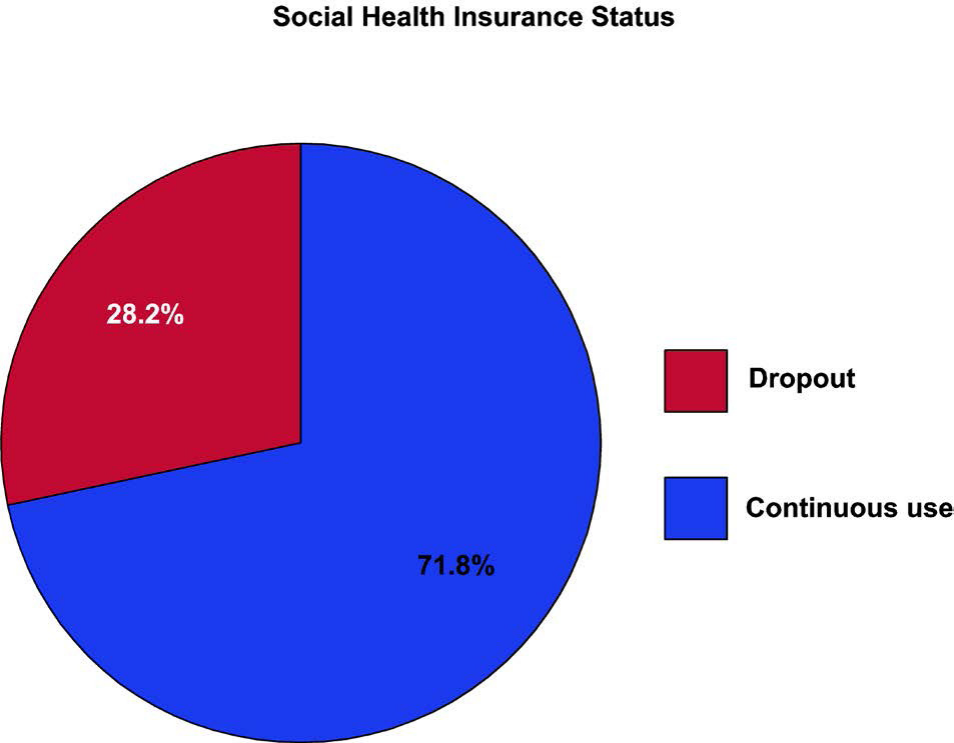
Social Health Insurance Dropout Status Among Households in Pokhara Metropolitan City, Kaski District, Nepal (n = 355).

The major reasons for dropping out from SHI included not having family consensus to renew (98%), long waiting time (63%), limited number of health facilities under SHI (60%), poor availability of drugs and services (34%), poor trust on SHI (36%), limited awareness on the renewal system (28%) and non-utilization of service benefits during the enrolment period (20%) (Table 3).

**Table 3:** Reasons for dropping out from SHI among households in Pokhara Metropolitan City, Kaski district, N epal (n= 255)

**Reasons**	**Number**	**Percent**
Information related		
Unaware of renewal system	28	28.0
Health service related		
Long waiting time	63	63.0
Limited health facility	60	60.0
No service and drugs available when needed	34	34.0
Unfriendly behaviour of service provider	16	16.0
Poor service quality	15	15.0
Limited scheme	11	11.0
Longer distance to health facility	10	10.0
Household related		
No family consensus	98	98.0
Unable to pay	6	6.0
Attitude related		
Poor trust on SHI	36	36.0
Services not used	20	20.0
No one got sick during enrolment period	9	9.0

Abbreviation: SHI, social health insurance.

###  Factors Associated With Dropout From SHI

Table 4 shows the factors associated with dropout from the SHI in bivariate and multivariate analyses.

**Table 4:** Factors Associated With Dropout From Social Health Insurance in Bivariate and Multivariate Analyses

**Explanatory Variables**	**uOR (95% CI) **	**aOR (95% CI)**
Household size		
>5 members	1.73 (1.08-2.78)	2.19 (1.22-3.94)^a^
≤5 members	Reference	Reference
Gender of household head		
Female	1.93 (1.20-3.11)	1.49 (0.79-2.80)
Male	Reference	Reference
Ethnicity		
Underprivileged ethnic group	1.74 (0.94-3.22)	2.36 (1.08-5.17)^a^
Privileged ethnic group	Reference	Reference
Home ownership		
Rented	3.12 (1.61-6.06)	4.53 (1.87-10.95)^b^
Self-owned	Reference	Reference
Presence of family member with chronic illness		
No	1.93 (1.20-3.12)	1.95 (1.07-3.59)^a^
Yes	Reference	Reference
Perceived health status of the family		
Good	3.02 (1.15-7.95)	4.21 (1.21-14.65)^a^
Poor	Reference	Reference
Health facility as a first contact point		
Private	2.17 (1.35-3.50)	3.75 (1.93-7.27)^b^
Public	Reference	Reference
Waiting time		
>60 min	0.24 (0.08-0.66)	0.34 (0.10-1.22)
30-60 min	0.48 (0.17-1.37)	0.87 (0.24-3.24)
<30 min	Reference	Reference
Availability of services under SHI		
Not available frequently	2.16 (1.24-3.77)	1.60 (0.78-3.28)
Frequently available	Reference	Reference
Availability of drugs under SHI		
Not available frequently	6.94 (2.10-22.99)	4.75 (1.19-18.95)^a^
Frequently available	Reference	Reference
Perceived behaviour of service providers		
Unfriendly	7.29 (3.31-16.02)	3.09 (1.01-9.49)^a^
Acceptable (average)	5.80 (2.49-13.50)	2.03 (0.77-5.36)
Friendly	Reference	Reference
Perceived quality of services		
Not satisfactory	2.61 (1.35-5.04)	1.75 (0.76-4.06)
Satisfactory	Reference	Reference
Satisfaction with opening hours of health facility		
Not satisfactory	2.62 (1.32-5.18)	1.26 (0.49-3.25)
Satisfactory	Reference	Reference
Affordability of premium		
Difficult to afford	1.92 (1.15-3.22)	1.37 (0.74-2.53)
Affordable	Reference	Reference

Abbreviations: uOR, unadjusted odds ratio; aOR, adjusted odds ratio; CI, confidence interval; SHI, social health insurance. ^a^ *P* < .05, ^b^*P* < .001; aOR is adjusted for all variables in the table.

The binary logistic regression, which was used to examine the independent association of explanatory variables with dropout status yielded a *P* value of less than 0.15 for fifteen explanatory variables. In the collinearity diagnostics, one explanatory variable indicated multicollinearity and was excluded from the analysis, while the remaining fourteen variables were subjected to multiple logistic regression.

 The results of the multivariate analysis showed that the odds of a household dropping out from SHI was significantly higher if their family size was more than five members (aOR: 2.19, 95% CI: 1.22-3.94) compared to those having five or less members. Similarly, the households from underprivileged ethnic groups (Dalit/Janajati) were 2.4 times more likely to drop out from SHI (aOR: 2.36, 95% CI: 1.08-5.17) compared to the ethnically privileged households. The odds that the households would drop out from the SHI was 4.5 times higher for families living on rented homes (aOR: 4.53, 95% CI: 1.87-10.95) compared to the ones who owned the houses by themselves. The households free from chronic illness had about two times higher odds of dropping out from the SHI program (aOR: 1.95, 95% CI: 1.07-3.59) compared to households having chronically ill family member. Likewise, the households that perceived the health status of their family as good were also about four times more likely to drop out from the SHI program (aOR: 4.21, 95% CI: 1.21-14.65) compared to those who perceived their family health status as poor.

The odds that the households would drop out from the SHI was four times higher for households which had private health facility as a first contact point (aOR: 3.75, 95% CI: 1.93-7.27) compared to their counterparts who had chosen public health facility. Similarly, the odds of dropping out from the SHI was higher for households that reported poor availability of drugs at the health facility (aOR: 4.75, 95% CI: 1.19-18.95) compared to those who reported that the drugs were frequently available. Furthermore, the households that perceived the behaviour of service providers as unfriendly had three times higher odds of dropping out from the SHI (aOR: 3.09, 95% CI: 1.01-9.49) compared to households who perceived the providers’ behaviour as friendly (Table 4).

## Discussion

This study mainly assessed the prevalence of dropout and associated factors among SHI enrolled households at Pokhara Metropolitan. In the current study, 28.2% of insured households dropped out of the SHI scheme. In the fiscal year 2018/2019, the HIB database had shown a dropout rate of 34.01% in the Kaski district.^4^ Whereas, another cross-sectional study from Pokhara metropolitan had found 55.6% dropout in the study population.^10^ In comparison to our findings, the figures from both studies are higher. Differences in dropout prevalence could be attributed to different study periods, sample sizes, and study sites.

The lack of availability of SHI-listed drugs in health facilities was the most significant factor influencing the households’ decision to drop out of the SHI scheme. Over the years, the HIB has substantially increased the number of items in the drugs list under the benefits package.^19^ Yet, the availability of these drugs at service delivery levels remains a longstanding concern.^33^ The absence of pharmacy services in the health facilities, limited public hospital budget and delayed procurement system could be some reasons why drugs listed in the benefits package are not procured timely and made available to the beneficiaries.^33,34^ The frequent stock-outs might have compelled the insured members to purchase the drugs from the open market. Having to pay for drugs through out-of-pocket despite having an active membership status might have discouraged the insured clients from continuing their SHI membership. Our findings corroborate those of prior research. According to the study by Nepal Health Research Council, the people were reluctant to become a member of the SHI scheme as the medicines and other services were not often available in the health facilities.^35^ Another explorative study of Nepal’s SHI scheme found that a lack of drugs and other supplies, particularly for chronic diseases patients, due to higher uptake of services and delay in procurement was the major determinant of poor enrolment.^4^ In sub-Saharan Africa, the unavailability of necessary drugs and providing single same drugs even for different illnesses that do not cure better was one of the important reasons why members were dissatisfied and dropped out from their health insurance scheme.^36^ This finding suggests the need for urgent efforts by the HIB to improve the purchasing and availability of drugs at service provider health facilities so as to restore the members’ trust and thus ensure their retention in the SHI scheme.

Home ownership is another significant factor influencing renewal or discontinuation of SHI membership. This study found that families living in rented homes were four times more likely to dropout than their counterparts who owned their own homes. One possible explanation could be the uncertainty among rented households about the continuation of their stay at their current location in the coming year. Those who are unsure how long they will stay or those who intend to leave their current location may have decided to withhold their health insurance plan. Despite the policy provision for households to change their first point of contact upon migration to other locations, the higher likelihood of dropping out by these households indicates that the households are unfamiliar with the practical details of the SHI provisions, including procedures for changing the first contact point. Because the Pokhara metropolitan city has a substantial proportion (55%) of families living in rented homes,^37^ greater attention and efforts towards rented households are deemed necessary. Providing clear awareness and information on the provisions of SHI, including policies and processes for changing first contact points, to rental households could be a useful strategy for guaranteeing their retention and increased coverage.

In the present study, the households that had chosen private health facility as a first point of contact had higher odds of dropping out from SHI scheme compared to their counterparts who had chosen public health facilities as first contact point. One possible reason behind this could be the removal of the private health facility by the HIB following the provision of the Health Insurance Act to only designate public health facilities as first contact point. Households with strong trust and preference towards private health sector thus might have decided to opt out of the SHI membership rather than selecting public sectors as their first contact point. On contrary, our findings could also be attributed to the members’ dissatisfaction with services of the private sectors. Numbers of anecdotal information reveals that private hospitals have been deceitfully deducting higher and unjustifiable amounts from the clients’ benefits package for utilization of low cost drugs and services.^38,39^ Also there are widespread concerns that private service providers have treated insured members as second-class citizens in comparison to their uninsured counterparts^34,40,41^ who often pay higher amounts through out-of-pocket. A similar case has also been reported in other countries’ health insurance schemes. For instance, in Thailand’s SHI scheme, some private providers were found to be skimping (using fewer resources to treat a patient than is necessary in order to increase profits) and dumping, resulting in a poor standard of care for insured patients.^42^

Unfriendly behaviour of service providers strongly predicted SHI dropout in our study population. The poor reception, lack of consideration, respect and attention for patients, rude approach, and not providing adequate and appropriate health information as well as unequal treatment to insured and uninsured members are often the key reasons why members are dissatisfied with the services^4,34,36,43^ and tend to opt out of the health insurance scheme. In a study about a Maliando Mutual Health Organization- a community health insurance scheme in West Africa, Criel and Waelkens found that the unpleasant behaviour of service provider was one of the important factors for dissatisfaction with the scheme and despite much higher charges, the members preferred to seek care from other private sectors and hospitals at their own expense ^36^. Another qualitative study from Ghana also revealed dissatisfaction with the health providers’ behaviour as one of the barriers for enrolment and retention of membership in the national health insurance scheme.^25^

This study also found an association between ethnicity and SHI dropout. When compared to ethnically privileged households, households from underprivileged ethnic groups were about three times more likely to drop out of the SHI. We attribute this finding to the underprivileged households’ precarious financial situation. In Nepal, Dalits and Janajatis make up a significantly larger proportions of the poor.^44^ Spending their limited earnings on insurance renewal is likely to jeopardize their ability to meet other pressing needs.^11^ Thus, maintaining financial protection against the uncertain future risks of catastrophic health spending may not be a top priority for these low-income marginalized groups.

Furthermore, this study confirmed an association between household size and SHI dropout. Households with more than five members were twice as likely as those with five or fewer members to drop out of the SHI scheme. The positive relationship between larger family size and SHI dropout could be due to a change in payment rates, resulting in an increased financial burden for premium payments. Due to the increase in annual premium rates (following the endorsement of the health insurance regulations), families renewing their insurance policies were required to pay much higher amounts than they had been paying previously. Additionally, the revised rate increased disproportionately for the larger household size. For instance, prior to the revision, the premium rate for the families of five, six and seven members was NPR 2500, NPR 3000 and NPR 3500 respectively. However, following the revision, the premium rates for families willing to join or renew their SHI scheme were raised to NPR 3500, NPR 4200 and NPR 4900 respectively. As a result of the inequitable increase, larger households may have been discouraged from maintaining their SHI membership. In study conducted in Burkina Faso, Dong et al had found that larger households are more likely to drop out of the CBHI scheme.^7^

More than a quarter of households dropping out from the SHI in this study stated lack of awareness regarding renewal mechanisms as their reason for not continuing the scheme. This suggests that over one-fourth of the dropouts in SHI could be averted simply by making the households informed about the renewal policies. It is a concerning fact that still a greater proportion of households are not aware of the renewal scheme while enrolment assistants are being mobilized by HIB at the each wards and communities to inform, convince and keep the families enrolled into the scheme.^17^ This point to the possibilities that the enrolment assistants might have become so much focused on increasing new enrolments that those already enrolled but requiring renewal may perhaps have received fewer reminder visits or no visits at all. The proactive involvement of enrolment assistants is therefore crucial to keep the insured families well informed of the renewal policy. Furthermore, HIB may also adopt digital innovations such as insurance renewal reminder service or text message notifications to inform households few weeks or months ahead of the subscriptions’ expiry. In Ghana, a mobile renewal application introduced in the National Health Insurance Scheme provides SMS reminder to health insurance members, including renewal of their membership and payment of premium.^45^

As in previous studies,^26,28^ our study also found that the absence of chronic illness in the family increased the probability for households not to renew their membership in a SHI scheme. In our view, it is reasonable to assume that the need for healthcare services for household without chronic illness are fewer and thus, they might be less concerned to continue their SHI membership.^27^ On the other hand, families with chronic illness require more frequent hospitals contacts and long-term medications intake. They might continue to remain insured in order to enjoy the benefits of the service package and to avoid paying out-of-pocket the high cost of healthcare and medicines. The greater tendency for households without pre-existing chronic health conditions to refrain from SHI membership might indicate the emergence of adverse selection. This is probable as the scheme in practice is not yet mandatory for all households. As greater proportion of healthier households drop out of the health insurance marketplace, more high-risk and sick households remain on the insurance pool. This is critical from the sustainability point of view, as the HIB will be forced to pay out a larger portion of healthcare claims in comparison to the premiums pooled by the program.^46^

It is believed that requiring mandatory enrolment in both the formal and informal sectors will help to avoid adverse selection problems. Nonetheless, even if SHI is mandatory, it is nearly impossible to force all people to join SHI, thus making the scheme de facto voluntary. As a result, the scheme continues to suffer from the same issues of low coverage, adverse selection, and fragmented risk pools.^47^ While SHI has been successful in achieving UHC in a number of high-income nations, attempt to replicate the similar models in low- and middle-income countries have been proven unsuccessful. For instance, Ghana’s mandatory national health insurance scheme which is often seen as a SHI success model,^48^ covers only 40% of the population.^49^ The contexts of many low- and middle-income countries are not conducive to expanding SHI coverage, as many informal sector workers and unemployed people are almost always excluded.^50,51^ Evidence suggests that all countries must adopt tax-based funding in order to achieve UHC. By funding UHC with tax revenues, a number of countries, including Sri Lanka, Malaysia, and Brazil, have demonstrated equitable healthcare access and UHC success.^52-54^ Importantly, the only low-income countries that have achieved universal and equitable health coverage have done so through taxation.^47^ Countries like Nepal should try to build on these countries’ UHC success stories by expanding free healthcare through tax revenue rather than premium contributions, as this may be a more promising and equitable path to universal access.

 Despite the important findings made in this study, it is admittedly fraught with certain limitations. First, a number of explanatory variables used in the study were related to the perceptions of the health insurance subscribers and there might be some bias because of the self-reported method used. Second, this study also does not distinguish the drop out between SHI members who have their premiums fully or partially subsidized by the government and those who pay the full premium. Moreover, because this is a cross-sectional study, we can only infer associations rather than causations. Despite these limitations, findings from this study may be useful to the HIB, policy-makers, service provider organizations and other stakeholders, including researchers who want improve the SHI coverage by addressing the low enrolment and higher dropout rates.

## Conclusion

 This study found a multitude of factors that influence households from dropping out of the SHI scheme. Having more than five members, belonging to underprivileged ethnic groups such as Dalit/Janajati, living on rented homes, absence of chronic illness in family, perceived good health status of the family, having private health facility as first contact point, not utilizing the service benefits, poor availability of drugs and perceived unfriendly behaviour of service providers are statistically significant factors associated with SHI dropout.

 We recommend that the HIB, and service provider health facilities and other stakeholders such as federal, provincial and local governments should provide more emphasis on addressing the problems of drugs availability and improving behaviour of service providers towards the insured members. Furthermore, increasing insurance awareness, including provisions to change first contact points, may aid in reducing dropouts among rented households, which account for a sizable proportion of the Pokhara metropolitan area. Besides, HIB also needs to adopt more pragmatic ways to ensure that low-risk households such as those with absence of chronic illness; with fewer needs to utilize health services and those perceiving their family health status as good, continue to remain in the scheme and enjoy better catastrophic financial protection. This may necessitate the enforcement of mandatory enrolment by all households, which is also necessary for the scheme to achieve its goal of achieving UHC. More importantly, the country should consider more efficient and equitable ways of raising revenue for health through tax reform. Increasing health insurance subscribers’ awareness of renewal policies and implementing insurance reminder notification systems may also aid in member retention in the SHI.

## Acknowledgements

 The authors would like to thank all the study participants for their participation and cooperation in the study. Amrit Raj Pokhrel, Rameshwor Baral, and Kapil Giri provided invaluable support in the data collection process. We acknowledge Pokhara Metropolitan for granting the permission to carry out this study and HIB, Gandaki province for providing the sampling frame. We particularly thank Shyam Thapa for his technical inputs and guidance throughout the research process. We are also grateful to Mridul Acharya for the editorial assistance.

## Ethical issues

 The study protocol was reviewed and approved by School of Health and Allied Sciences and ethical clearance was obtained from the Institutional Review Committee of Pokhara University. Prior approval to execute the study was obtained from Pokhara Metropolitan City and HIB, Gandaki province. Before the interview, the respondents were briefed on the study’s objectives as well as the benefits and risks of participating in the study and written informed consent was obtained. In addition, all information obtained from the respondents was kept confidential. The questionnaire did not include any names or other personal identifiers. Each participant’s identity was determined by numerical coding. The participants were informed of their right to refuse to participate in this study as well as their right to withdraw from the interview at any time.

## Competing interests

 Authors declare that they have no competing interests.

## Authors’ contributions

 PS designed the study, acquired and analyzed the data and drafted the manuscript. DKY and NS helped revise the study design, supervised the data analysis and conducted some part of the analysis. PG contributed in drafting and revising the manuscript as well as in the analysis and interpretation of data. All authors read and approved the final manuscript.
